# Local Wound Infiltration with Ropivacaine for Postoperative Pain Control in Caesarean Section

**DOI:** 10.7759/cureus.5572

**Published:** 2019-09-05

**Authors:** Fahmia Nasir, Irum Sohail, Hasina Sadiq, Maria Habib

**Affiliations:** 1 Obstetrics and Gynecology, KRL Hospital, Islamabad, PAK

**Keywords:** caesarean section, ropivacaine, analgesia

## Abstract

Background

The rate of the caesarean section has been on a progressive rise ever since its association with improved fetal prognosis was established. This study was conducted to assess the effect of local wound infiltration with ropivacaine on the postoperative analgesia requirement and pain scores in caesarean section patients.

Methods

This was a prospective single-blinded randomized control trial conducted at the Department of Obstetrics and Gynecology, KRL Hospital, Islamabad, Pakistan over a duration of six months from January 2018 to June 2018. All the women aged 19 to 40 years, who underwent elective caesarean sections under spinal anesthesia, with American Society of Anesthesiologists (ASA) score II, were included in the study and randomized into two groups. The primary outcome studied was the efficacy of ropivacaine in controlling postoperative wound pain compared to no local analgesic. Pain severity was assessed using the visual analog scale (VAS) which was explained to the patient beforehand and which comprised a range of score from zero (no pain) to 10 (worst pain imaginable). Initially, paracetamol 1 g intravenous (IV) was given every six hours, over 24 hours. If pain did not settle on this, ketoprofen 3 mg/kg IV was given every eight hours, and in case of further analgesic demand by the patient, nalbuphine 10 mg was given IV, if necessary. The data was collected on a specific questionnaire and analyzed on the Statistical Package for Social Sciences (SPSS Inc., Chicago, IL) version 23.0. A p-value of less than 0.05 was considered significant.

Results

A total of 100 patients were randomized into two groups. Pain scores were significantly reduced in the ropivacaine group at four, six, and 12 hours after surgery. The percentage of patients who requested the multiple doses of IV paracetamol, ketoprofen, and nalbuphine, was significantly lowered in the ropivacaine group as compared to the placebo group (p<0.001).

Conclusions

Local infiltration with ropivacaine during caesarean section significantly reduces the postoperative analgesic requirement and visual analog scores, reducing the incidence of side effects.

## Introduction

The rate of the caesarean section has been on a progressive rise ever since its association with improved fetal prognosis was established [1]. Worldwide, around 15% to 20% of the deliveries are performed through caesarean sections and the rate is even higher in developing countries where it is estimated to be about 40% [2]. Postoperative pain is one of the most preventable adverse outcomes of surgery and is believed to be due to the physiological response of tissue injury at the site of surgery [3]. According to a survey done in America in 2003, there is a high chance of approximately 70% that a patient will experience severe pain postoperatively [4]. Effective postoperative pain management is important as it has been shown to be closely related to patient’s recovery, length of hospital stay, stress response, and complications like pneumonia, deep venous thrombosis, poor wound healing, chronic pain, and depression [3,5].

Nowadays multimodal analgesia, which means the use of combination drugs for pain relief with decreased side effects, is in practice [6]. Opioids are the first-line drugs to relieve postoperative pain following caesarean sections, either administered via intrathecal route prior to surgery or intravenous (IV) route postoperatively [1]. However, at the same time, their use has been associated with complications such as respiratory depression, urinary retention, pruritis, ileus, nausea, and vomiting following surgery [3]. On the other hand, non-opioid systemic analgesics have failed to be adequate in relieving pain, and other methods like epidural analgesia require vigilant monitoring and surveillance [1,3].

Recently preventive analgesia has been introduced, which means intraoperative or postoperative analgesic intervention, is capable of reducing the postoperative pain and further drug consumption [7]. Various forms are available like transverses-abdominis plane block, IV, and local anesthetic infusion [3]. Wound infiltration with local anesthetics is being widely introduced into clinical practice due to its simplicity, safety, and cost-effectiveness [5]. The aim of this study is to establish the efficacy and safety of local infiltration of 7.5 mg/ml of ropivacaine as an analgesic in patients undergoing caesarean section by analyzing the total consumption of opioid and non-opioid systemic analgesics in first 24 hours following surgery. Ropivacaine acts by inhibiting the transmission of noxious stimuli from the site of injury by reversible hyperpolarization of peripheral nerve fibers and hence reduces the opioid consumption [8]. This study can open new dimensions in the management of pain in the postoperative period as all such advances are the need of the hour.

## Materials and methods

This study was a prospective single-blinded randomized controlled trial conducted in the Department of Obstetrics and Gynecology, KRL Hospital, Islamabad, Pakistan over a period of six months duration from January 2018 to June 2018. It was conducted after the approval of the hospital’s ethical committee and patients were prospectively enrolled in the study. This was all managed and followed by the same resident doctors in the operation theater and intensive care units for postoperative care. Prior to being enrolled, all patients were well-informed about the purpose and method of the study and only those patients were included who gave written informed consent. All the women aged 19 to 40 years, who underwent elective caesarean sections under spinal anesthesia, with American Society of Anesthesiologists (ASA) score II, were included in the study. Emergency caesarean sections, use of general or epidural anesthesia, contraindication to the use of non-steroidal anti-inflammatory drugs (NSAIDs), history of cardiopulmonary disorders, allergy to the study drugs, drug dependence or history of chronic and neuropathic pain syndrome led to exclusion of the patients from the study.

Randomization was performed using random number allocation and patients were divided into two groups, group R (ropivacaine group) and group P (placebo group). All participants received spinal anesthesia under ASA grade II, were operated upon by the same surgical team under standard circumstances so that the bias could be alleviated, and received Pfannenstiel incision with a peritoneal opening. At the time of skin closure, ropivacaine 5 mg/ml (diluted to 20 ml) or 0.9% normal saline 20 ml was infiltrated in the subcutaneous tissue. Half of the volume (10 ml) was injected at the upper edge and remaining half at the lower edge of the incision. Patients were not aware of the group to which they belonged.

The primary outcome studied was the efficacy of ropivacaine in controlling postoperative wound pain compared to the placebo group. Pain severity was assessed using the visual analog scale (VAS) which was explained to the patient beforehand and which comprised a range of score from zero (no pain) to 10 (worst pain imaginable). VAS scoring was done by a resident doctor at two, four, six, and 12 hours after caesarean section. Postoperative analgesics were administered either on the patient’s demand or if the VAS was greater than four. Initially, paracetamol 1 g intravenous (IV) was given every six hours, over 24 hours. If pain did not settle on this, ketoprofen 3 mg/kg IV was given every eight hours, and in case of further analgesic demand by the patient, Nalbuphine 10 mg was given IV if necessary.

The secondary outcomes were the total amount of analgesic administered to the patient in the first 12 hours, any complications like respiratory depression, nausea, vomiting, paralytic ileus or pruritis, and time to bowel sounds. Patients were also followed for any drug-related complications.

All the data was analyzed on the Statistical Package for Social Sciences (SPSS Inc., Chicago, IL) version 23.0. The qualitative variables were analyzed by using the chi-square test. Means for quantitative variables like age, weight, pain scores, duration of anesthesia, duration of surgery, and time to first analgesic demand were compared by independent samples t-test. A p-value below 0.05 was considered statistically significant.

## Results

All the patients completed the study and successfully underwent a lower segment caesarean section including wound infiltration with a prearranged solution. Parturients in the two groups were comparable in terms of age, weight, duration of surgery, and duration of anesthesia as shown in Table 1.

**Table 1:** Basic demographic profile SD: Standard deviation.

Parameters	Ropivacaine Group (Mean ± SD)	Placebo Group (Mean ± SD)	p-Value
Age (years)	29.48 ± 4.76	30.46 ± 5.66	0.068
Weight (kilogram)	76.40 ± 12.4	75.90 ± 14.2	0.084
Duration of Anesthesia (minutes)	37.30 ± 6.07	36.70 ± 5.49	0.388
Duration of Surgery (minutes)	37.70 ± 6.24	36.70 ± 5.49	0.240

Pain scores were significantly lower in the ropivacaine group at four, six, and 12 hours after surgery as shown in Table 2 but insignificant among both groups at two hours post-caesarean that was probably due to the effect of spinal anesthesia. The time until the first analgesic demand was longer in the ropivacaine group.

**Table 2:** Mean pain scores and time to first analgesia demand VAS: Visual analog score; SD: Standard deviation.

VAS Scores	Ropivacaine (Mean ± SD)	Placebo (Mean ± SD)	p-Value
VAS at 2 Hours	1.12 ± 1.08	4.88 ± 1.88	0.306
VAS at 4 Hours	2.14 ± 1.10	5.38 ± 1.65	<0.001
VAS at 6 Hours	1.98 ± 1.13	4.38 ± 1.95	<0.001
VAS at 12 Hours	1.86 ± 1.34	2.60 ± 2.28	<0.001
Time to First Analgesic Demand (Hours)	4.49 ± 1.12	2.36 ± 0.58	0.007

Our study showed that among the patients who requested for the postoperative analgesia, about 82% of patients in the placebo group required three doses of paracetamol as compared to 44% of patients in the ropivacaine group. Similarly, 14% of patients in the placebo group required three doses of nalbuphine as compared to 10% of patients in the ropivacaine group who required only two doses and none of them required three doses. The results are shown in Table 3.

**Table 3:** Frequency of postoperative systemic analgesics in both groups

Systemic Analgesic	Doses	Placebo		Ropivacaine		p-Value
		Frequency	Percentage	Frequency	Percentage	
Paracetamol	One Dose	0	0%	3	6%	<0.001
	Two Doses	9	18%	25	50%	
	Three Doses	41	82%	22	44%	
Ketorolac	One Dose	0	0%	9	18%	<0.001
	Two Doses	29	58%	40	80%	
	Three Doses	21	42%	1	2%	
Nalbuphine	One Dose	9	18%	45	90%	<0.001
	Two Doses	34	68%	5	10%	
	Three Doses	7	14%	0	0%	

In both groups, the patients were monitored for the presence of nausea and vomiting, paralytic ileus, and pruritis during the first 24 postoperative hours and the findings were recorded on a chart. No case of nausea, vomiting, pruritis or ileus was reported in the study. The urinary catheter was removed after 12 hours of surgery and normal bladder function resumed with no cases of urinary retention noted amongst the participants.

## Discussion

Wound infiltration with local anesthetic is an addition to multimodal analgesia approach for postoperative pain relief in a number of surgeries including breast, hernia repair, and laparoscopic procedures [5]. Mainly intermediate and long-acting anesthetic agents are used like ropivacaine and bupivacaine. Ropivacaine is less lipophilic than bupivacaine, therefore, has a significantly higher threshold for cardiotoxicity and neurotoxicity than bupivacaine [9]. That is the reason for it being widely used as a long-acting regional anesthetic while reducing the potential toxicity and improving relative sensory and motor block profiles simultaneously. Previous studies have demonstrated that local anesthetic infiltration was effective in controlling postoperative pain.

Nguyen et al. conducted a study determining the efficacy of Pfannenstiel incision infiltration with 7.5 mg/ml ropivacaine for a caesarean section in 2010 by dividing the patients into the ropivacaine group and control group. He concluded that this intervention led to a reduction of 30% in the overall consumption of analgesics, especially opioids in the first 24 hours following surgery. It also significantly increased the time until the first analgesic request by the patient by two hours and 26 minutes. There was no significant difference in the threshold of VAS in the two groups [1].

Another study compared epidural morphine and continuous caesarean wound infusion with ropivacaine for 48 hours postoperatively for pain relief and side effects relief. A total of 58 women were randomly allocated to one of the two respective groups. The results were in favor of the ropivacaine group as better pain relief, lesser side effects, lesser nursing care, and shorter hospital stay was seen (p<0.001) [10]. This was consistent with the results of our study. Ropivacaine has also been used as analgesia for post-caesarean pain relief via the patient-controlled elastomeric pump. The results show better pain relief and therefore lesser opioid consumption and better patient comfort in the postoperative period [11].

Several studies have been conducted comparing the local infiltration of bupivacaine with placebo or other drugs like tramadol for post-caesarean pain relief. One of the studies conducted in Iran by Sahmeddini et al. compared local infiltration of bupivacaine and tramadol. This study concluded no significant difference in pain relief until 16th postoperative hour but after that, the tramadol was statistically significant in reducing the pain after the 16th hour. No significant difference in side effects like postoperative nausea, vomiting, and respiratory depression was observed [12].

In another study conducted in India, Sachidananda et al. compared bupivacaine with a mixture of bupivacaine and tramadol for post-caesarean pain relief. They showed that time for the first analgesic request was delayed in the mixture group compared to the bupivacaine group alone. Additionally, the total diclofenac consumption in the first 24 hours was also reduced in the mixture group and patient satisfaction was more in this group [13]. A local comparative study conducted in Karachi, Pakistan in which two groups; one study group with local infiltration of bupivacaine and another control group, were taken. The results showed that the additional analgesia like parenteral tramadol requirement was more in the control group (p<0.001). No major side effects were observed in the bupivacaine group [14].

Another study conducted by Sarwar et al. in Pakistan compared mean narcotic analgesia requirement in patients undergoing caesarean section with and without bupivacaine infiltration. The results showed a significantly lowered mean pain score of 1.98 ± 0.91 in bupivacaine group whereas a mean pain score of 2.8 ± 1.23 in the non-bupivacaine group. While at the same time, mean postoperative narcotic analgesia requirement of 32 ± 51.27 mg in the bupivacaine and 82 ± 84.97 mg in the non-bupivacaine group, with a significant p-value of less than 0.001 was shown. Hence the study revealed that patients with bupivacaine infiltration required significantly lower doses of narcotic analgesia [15].

The limitation of our study included decreased time duration and a small study population.

## Conclusions

This study concludes that the use of ropivacaine for local wound infiltration at the time of the caesarean section reduces the overall consumption of systemic analgesics in the postoperative period by reducing the pain scores. This also delays the patient’s on-demand analgesic by more than four hours and hence increases the pain-free interval following surgery.
